# A class of DNA-binding peptides from wheat bud causes growth inhibition, G_2 _cell cycle arrest and apoptosis induction in HeLa cells

**DOI:** 10.1186/1476-4598-8-55

**Published:** 2009-07-31

**Authors:** Loretta Mancinelli, Paula M De Angelis, Lucia Annulli, Valentina Padovini, Kjell Elgjo, Gian Luigi Gianfranceschi

**Affiliations:** 1 Department of Cellular and Environmental Biology, CEMIN (Center of Excellence on Innovative Nanostructured Materials for chemical, physical and biomedical applications), University of Perugia, via Pascoli 06123 Perugia, Italy; 2 The Pathology Clinic, Oslo University Hospital, N-0027 Oslo, Norway

## Abstract

**Background:**

Deproteinized DNA from eukaryotic and prokaryotic cells still contains a low-molecular weight peptidic fraction which can be dissociated by alkalinization of the medium. This fraction inhibits RNA transcription and tumor cell growth. Removal from DNA of normal cells causes amplification of DNA template activity. This effect is lower or absent in several cancer cell lines. Likewise, the amount of active peptides in cancer cell DNA extracts is lower than in DNA preparation of the corresponding normal cells. Such evidence, and their ubiquitous presence, suggests that they are a regulatory, conserved factor involved in the control of normal cell growth and gene expression.

**Results:**

We report that peptides extracted from wheat bud chromatin induce growth inhibition, G_2 _arrest and caspase-dependent apoptosis in HeLa cells. The growth rate is decreased in cells treated during the S phase only and it is accompanied by DNA damage and DNA synthesis inhibition. In G_2 _cells, this treatment induces inactivation of the CDK1-cyclin B1 complex and an increase of active chk1 kinase expression.

**Conclusion:**

The data indicate that the chromatin peptidic pool inhibits HeLa cell growth by causing defective DNA replication which, in turn, arrests cell cycle progression to mitosis via G_2 _checkpoint pathway activation.

## Background

After intensive deproteinization with NaCl, SDS, chloroform/isoamyl alcohol and phenol the DNA preparation from eukaryotic or prokaryotic cells still contains a low molecular weight peptidic material [1]. By further treating this preparation with alkaline buffer it is possible to extract a pool of peptides (20 μg/mg of DNA), with a molecular weight of about 1000 Da, consisting of sequences with strongly related amino acid composition [2]. Its binding to DNA is pH dependent and decreases from pH 7.5 to 9.5 [3]. Using such a procedure of extraction, this family of peptides was found in the chromatin of eukaryotic (calf thymus, bull spermatozoa, trout testis, pea bud and wheat germ) [4-6] and prokaryotic (E. coli and lambda phage) cells [7,8]. They do not seem to be the products of proteolytic degradation occurring during the extraction since the same peptide fraction is obtained if a cocktail of protease inhibitors is added to all the solutions used [9].

Independently of the source of extraction, their biological effects are quite similar. It has previously been reported that these peptides inhibit RNA transcription in cells and in vitro reconstituted systems with prokaryotic and eukaryotic RNA polymerase [10,11], and that they stabilize the structure of double-stranded DNA, thus increasing its melting point [12]. When they are removed by alkaline buffer from DNA of normal cells DNA template capacity is enhanced [13] but this effect is lower or even absent in several cancer cell lines [14]. Transcriptional data are in agreement with the melting profile of DNA from cancer cells which show no significant differences between acid and alkali extracted DNA. Accordingly, the level of active peptide fraction extracted from the DNA of cancer cells is markedly lower than that obtained from DNA of the corresponding normal cells [15]. We have also found that the peptide fraction is able to decrease the growth rate of HL60 and L1210 tumour cell lines [16]. The biological effects of this class of peptides and their ubiquitous presence in prokaryotic and eukaryotic cells indicate that they could be involved in a conserved mechanism of cell growth control and gene expression. Their low concentration in cancer cells DNA as compared to normal cells indicates that this putative control mechanism may be lost during carcinogenesis. It is, therefore, of interest to investigate their potential role in controlling cell proliferation.

In this paper we report the activity of DNA peptides, extracted from wheat buds, in inhibiting the growth of HeLa cells. In order to gain more knowledge about their molecular mechanism of action, apoptosis induction and the effects on cell cycle progression were also investigated.

## Results

### Chromatin peptide fraction inhibits cell growth

**Figure 1:**
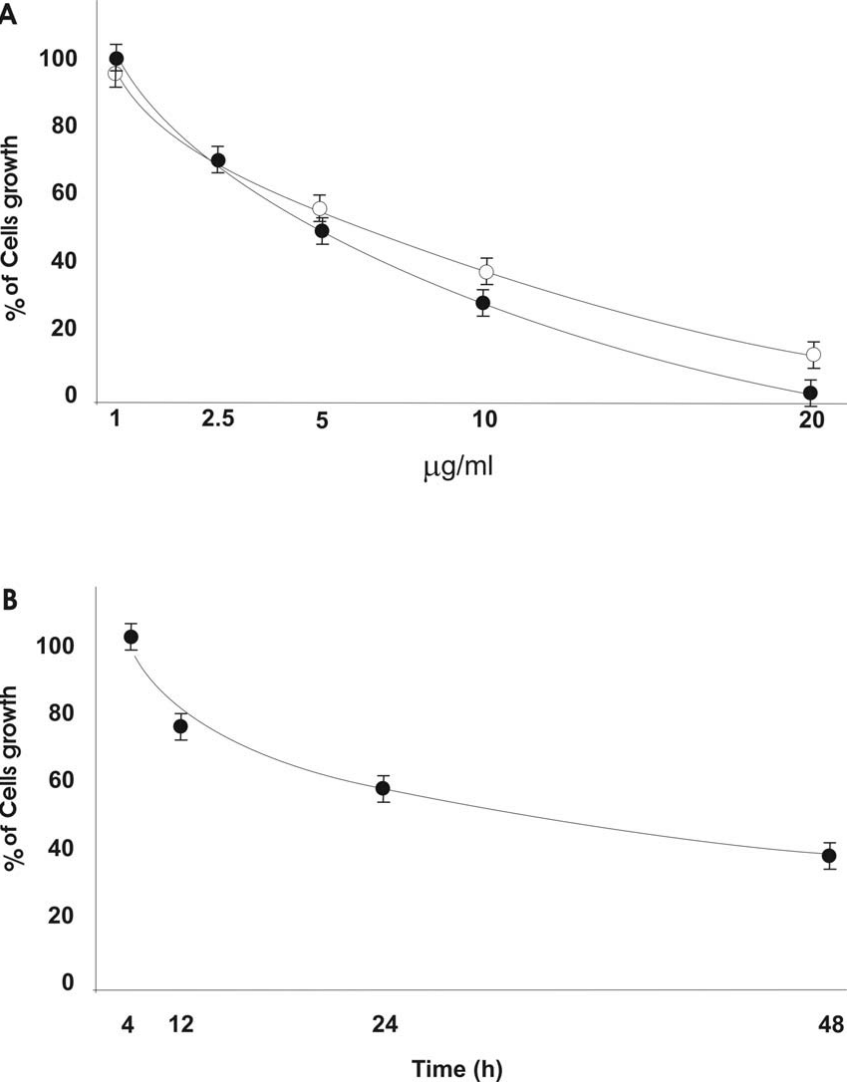
**Cell growth following incubation with the peptide fraction**. The cells were incubated for 24 hours (white circles) and 48 hours (black circles) with different concentration of the peptidic pool (A) and for different times with 5 μg/ml of the peptide fraction (B). The results are expressed as percentage of cell number in treated cultures.

HeLa cells were incubated with different concentrations of the chromatin peptide fraction for 24 and 48 h and the cell number was determined by the trypan blue dye exclusion method (Fig. 1A). The concentration (5 μg/ml) that reduced the number of the cells by approximately 40% with respect to the controls after 24 h of incubation, was tested further at different time points (Fig. 1B). The peptide fraction inhibits the proliferation of HeLa cells in a concentration and time dependent manner. The growth inhibition induced did not seem to be due to general toxicity because the number of dead cells in treated and control cultures was the same (about 5%) as revealed by the trypan blue exclusion assay. Moreover, in order to exclude toxicity, the activity of the lactic dehydrogenase enzyme in the culture medium was determined. It was undetectable even in cell cultures incubated with high concentration of chromatin peptides where the inhibition was about 80%. In all the subsequent experiments the cells were treated with 5 μg of peptide fraction per ml of culture medium. Since the peptide sequence effector/s has not been isolated, this concentration represents the total amount of the peptides constituting the pool.

### Chromatin peptide fraction induces apoptosis

**Figure 2:**
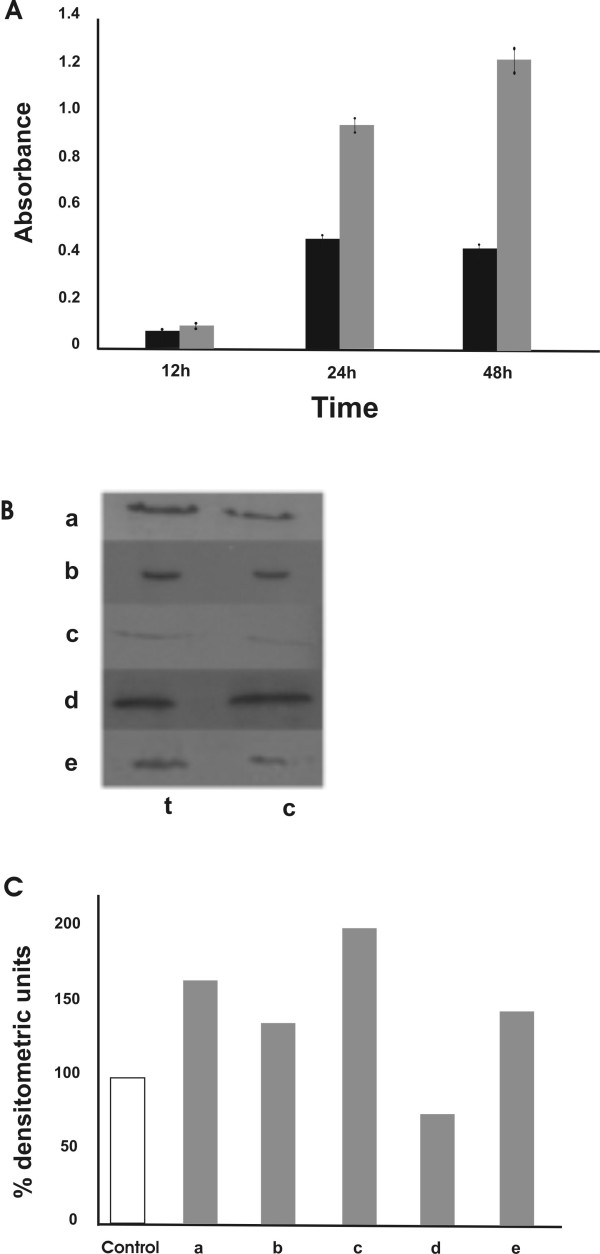
**Chromatin peptides induce apoptosis in HeLa cells**. A: apoptosis level measured by DNA fragmentation ELISA after 12, 24 and 48 hours of treatment with 5 μg/ml of peptide pool. (black bars) control cells; (grey bars) treated cells. B: analysis of proteins involved in apoptosis induced by chromatin peptides. Whole cell extracts were prepared from treated (24 h) and untreated HeLa cells. The same amount of proteins was assayed by PAGE and western blotting using antibodies specific for: (a) cleaved caspase-7, (b) cleaved caspase-9, (c) Bax, (d) Bcl-2 and (f) cleaved PARP. C: densitometric analysis of the immunoblots. The values are expressed as percentage of the control.

Apoptosis levels were measured by using a DNA fragmentation ELISA test in cells incubated for 12, 24 and 48 h (Fig 2A). Apoptosis induction was also quantified by FACS using a terminal (TαT) deoxynucleotidyl transferase assay as described in Materials and Methods. After 24 h of incubation with the same peptide concentration the percentage of apoptotic cells was about 30% higher than that found in untreated cultures. In order to investigate the apoptotic pathway, the inactivation of PARP, the induction of caspase cleavage and the involvement of Bcl-2 family proteins were analyzed. Figure 2B shows that the peptide treatment induced the cleavage of caspases 7 and 9 (lines a and b), PARP inactivation (line f), a higher expression of the pro-apoptotic protein Bax (line c) and a decreased level of the anti-apoptotic Bcl-2 protein (line d) (the densitometric values are reported in Figure 2C). These data suggest that the apoptosis process induced by chromatin peptides is caspase dependent and involves the mitochondria.

### Effect of chromatin peptides on cell cycle progression

**Figure 3:**
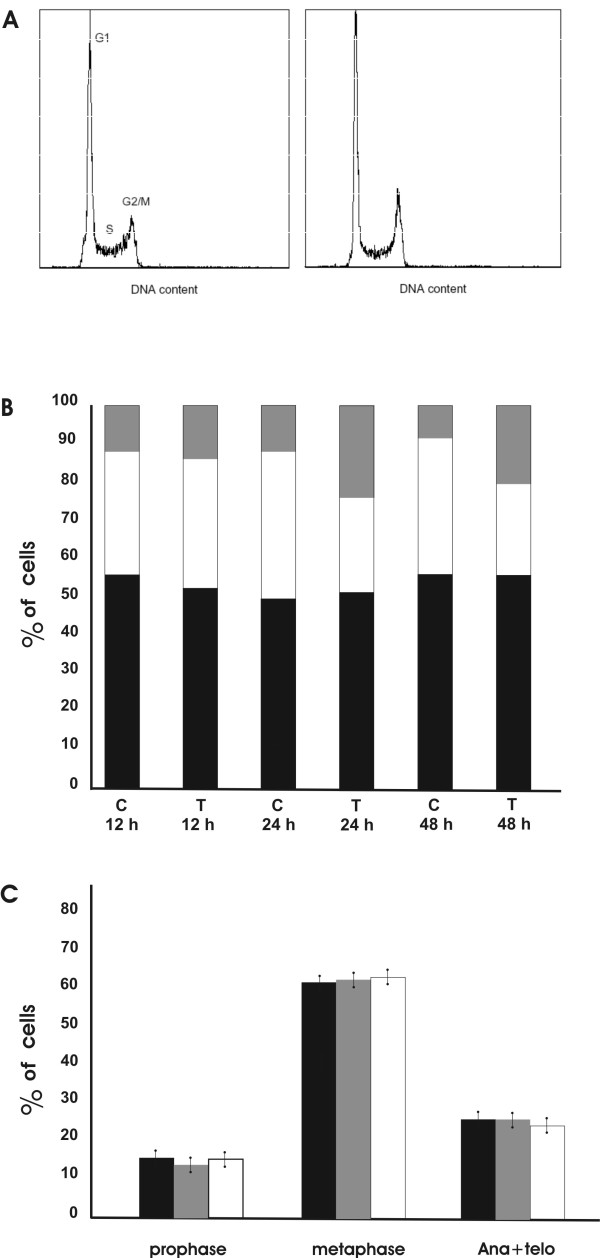
**G_2 _arrest of HeLa cells following the treatment with the peptidic pool**. A: DNA distributions for control (left) and treated (right) HeLa cells after treatment with 5 μg/ml of the peptide pool for 12 h. B: Cell cycle changes after 12, 24 and 48 hours of incubation with 5 μg/ml of peptide pool. Percentage of cells in: (black) G_1 _phase; (white) S phase; (grey) G_2_/M phase; c: control cells; t: treated cells. C: Distribution of the cells in the mitosis phases following incubation for 24 hours with (grey bars) 2.5 μg/ml or with (white bars) 5 μg/ml of peptides; (black bars) control cells.

To determine more precisely how the chromatin peptide fraction inhibits HeLa cell proliferation, we investigated whether the apoptotic process was accompanied by a cell cycle perturbation. Flow cytometry analysis was performed on cells treated for 12, 24 and 48 h with 5 μg/ml of chromatin peptides to assess the distribution of cells in the various phases of the cell cycle. As shown in Fig. 3A and 3B the treatment caused an increase in the percentage of cells in G_2_/M phase that was accompanied by a decrease of the cell number in S phase. Coefficients of variation (CVs) for the G_1 _peaks in the cell cycle distributions were 5%. The flow cytometry data demonstrate that the chromatin peptides arrest the cells in G_2 _or early M phase. It is known that G_2 _arrest is induced to prevent transition to mitosis in the presence of DNA damage [17], while early mitotic arrest is due to the action of checkpoints that ensure that the cells do not exit mitosis until the genome has been properly segregated. In order to study the mechanism by which the chromatin peptides affect cell cycle progression, we examined the position in the cell cycle of the arrested cells. As shown in Fig 3C, the numbers of the cells in prophase, metaphase and anaphase + telophase were not modified by the treatment. This suggests that the treated cells do not enter mitosis and that chromatin peptides affect the cell cycle of HeLa cells by inducing an arrest in G_2 _phase. We therefore investigated the possibility that this cell-cycle block could result from DNA damage induced by the action of this peptide pool.

### Selective effect of the chromatin peptides on S phase cells

**Figure 4:**
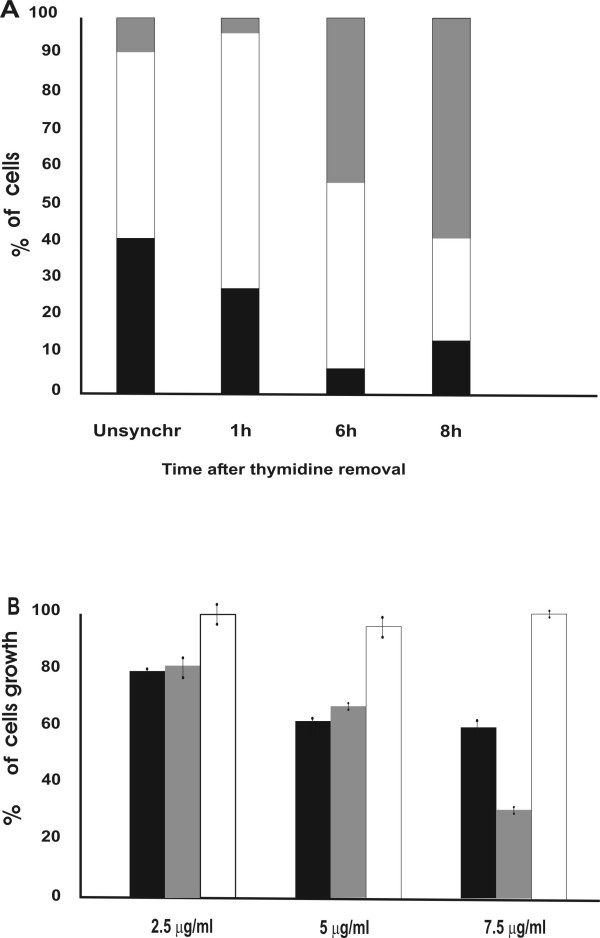
**Effects of the chromatin peptides on the various phases of the cell cycle**. A: Cell cycle analysis by FACS of unsynchronized cells and of synchronized cells at 1, 6 and 8 hours after removal of the thymidine block. (black) percentage of cells in G_1 _phase; (white) percentage of cells in S phase; (grey) percentage of cells in G_2_/M phase. B: Cell growth after treatment with different concentration of peptide fraction in the various phases of cell cycle as described in the text; (black bars) treatment during S phase; (grey bars) treatment during S+G_2 _phases; (white bars) treatment during G_2 _phase as described in the text.

Previous studies have shown that the chromatin peptides can inhibit DNA transcription. Moreover, once extracted from DNA, they can rebind to it in the presence of divalent cations. Consequently the action of these peptides in modifying cell proliferation and gene expression could possibly be a result of their effects on DNA activity. In accordance with this hypothesis we examined whether treatment with the peptide fraction has a selective effect on the S phase of the cell cycle. The antiproliferative effect was therefore evaluated by treating synchronized HeLa cells during each single phase. Synchronization was performed by the double thymidine block that arrests the cells at the G_1_/S boundary, while the removal of thymidine induces the onset of S phase [18]. The duration of S phase was examined by determination of ^3^H-thymidine uptake into DNA immediately after removal of thymidine block, at 1 hour intervals. The time course of thymidine incorporation (data not shown) indicates that uptake of ^3^H-thymidine initiated 1 h after the removal of the thymidine block, reached a maximum after 6 h and dropped to the initial level after 8 h. The cells were also counted to establish the mitosis time point. Their number remained constant for nine hours after the release of the block. At 11 h the number of the cells doubled indicating that cell division was completed. The position of the cells in the cell cycle was assessed by flow cytometry analysis performed at 1 h, 6 h and 8 h after removal of the thymidine block (Fig. 4A). In order to verify whether the synchronization method interferes with the antiproliferative action of the chromatin peptides, synchronized cells were incubated for the whole cell-cycle (23 h) with different concentration of the peptide pool. A dose dependent inhibition of cell growth was obtained also after synchronization and its level was comparable to that reported for unsynchronized cells (data not shown). The treatment in S phase was carried out by incubating the cells with the peptide fraction for 3 hours starting at 3 h after removal of the thymidine block. Then the medium containing the peptide fraction was replaced with normal medium and the cells were counted at the end of M phase. The inhibition was calculated as percentage of the control cells grown in the same way.

The treatment during S phase induced a dose-dependent inhibition, while no differences in cell number were obtained when the cells were incubated with the peptide fraction during the G_2 _phase. No further increase in growth inhibition was obtained when the treatment was carried out for the period corresponding to S + G_2 _phases except when a high dose of peptides was used (Fig. 4B). The treatment during G_1 _phase was performed in HeLa cells synchronized by the serum starvation method. With this method the replacement with the normal medium releases the cells at the G_0_/G_1 _boundary [19]. The peptide fraction was added at this time point and the cells were counted after 23 hours of treatment. In this situation the percentage of growth inhibition is about 38%. No growth inhibition was obtained when the cells were incubated with the peptide fraction during G_1 _phase only (15 hours of treatment) and allowed to grow in normal medium until the end of mitosis (data not shown). These data indicate that the major effect of cell growth inhibition by chromatin peptides is obtained when cells are treated in S phase. It suggests that their effect on cell growth is exerted by targeting a mechanism specific to S phase. To support this hypothesis we tested the effect of the chromatin peptide fraction on DNA synthesis. At 6 hours after the removal of the double thymidine block,^3^H-thymidine incorporation in the presence of chromatin peptides showed about 45% inhibition as compared with the controls. This result is consistent with the reported sensitivity of the S phase cells to the chromatin peptide treatment and with the hypothesis that they might exert their inhibitory effect on cell growth by affecting DNA synthesis.

### DNA damage induced by chromatin peptides

**Figure 5:**
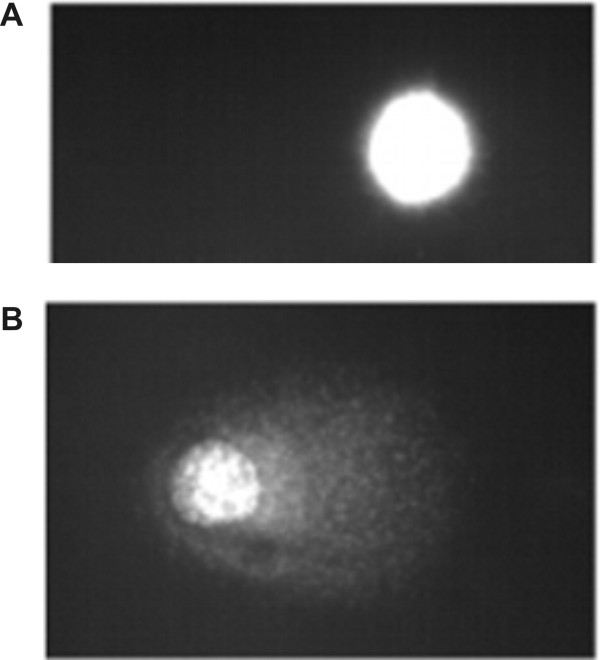
**Image of DNA migration obtained by fluorescence microscopy following single cell electrophoresis performed on S phase cells**. A: control cells; B: cells incubated for 2 hours with 5 μg/ml of peptide fraction.

It has earlier been reported that the chromatin peptide fraction binds to DNA and inhibits RNA synthesis and DNA replication in cells and in cell free systems [6,11,20]. Here we show that the treatment decreases DNA synthesis in the S phase of HeLa cells indicating that these peptides interfere with the DNA template capacity. It is thus reasonable to hypothesize that DNA damage could be induced by the treatment. DNA damage was therefore checked by exposing S phase cells to the chromatin peptides at 1 h after the removal of the thymidine block for 2 hours. They were harvested and subjected to Comet assay under different pH conditions. When the cells are lysed and subjected to electrophoresis under neutral conditions only double strand breaks are detected. Under alkaline conditions, because of DNA denaturation, single strand breaks are also detected [21]. The peptide treatment induces increased DNA migration only in the alkaline Comet assay (Fig. 5). The tail moment, expressed as Arbitrary Units, was in the range of 4–14 for control cells and 44–82 for treated cells. These data indicate that DNA damage results in the formation of single strand breaks and support the hypothesis that DNA template activity is the target for the chromatin peptides. The first step of their action could be inhibition of DNA synthesis.

### Chromatin peptides cause inactivation of CDK1-cyclin B1 complex

**Figure 6:**
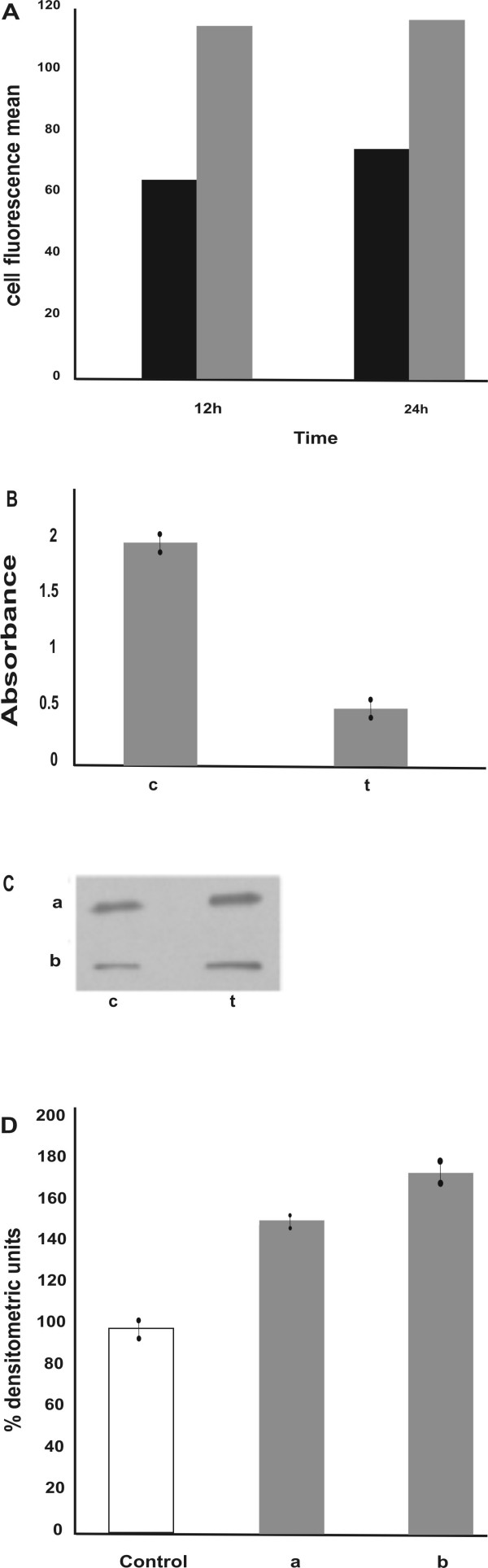
**Induction of the G_2 _checkpoint control by chromatin peptides**. A: Expression of cyclin B1 at 12 and 24 hours of peptide fraction treatment. (black bars) control cells; (grey bars) treated cells. The analyses were performed by flow cytometry as described in Materials and Methods. B: Cdc2-cyclin B1 activity determined by ELISA in nuclear extracts obtained from G_2 _cells. C: Western Blot analysis performed in nuclear extracts obtained from G_2 _cells. a: inactive phospho-Tyr15 cdc2-cyclin B1 complex; b: active phospho-Ser345 chk1. D: densitometric analysis of the immunoblotting. The values are expressed as percentage of the control. c: control cells; t: treated cells.

It is well known that eukaryotic cells arrest in G_2 _in response to DNA damage during S phase. This cell cycle arrest is crucial to avoid entry into mitosis of cells with genomic defects. The G_2_/M transition is under the control of the CDK1 kinase whose activity depends on its association with the regulatory subunit cyclin B1. In cycling cells, cyclin B1 synthesis begins in late S-early G_2 _phase. The formed complex is not active because of inhibitory phosphorylation at Thr14 and Tyr15 by Myt 1 and wee1 kinases [22]. In order to study the mechanism by which HeLa cells arrest in G_2 _after peptide fraction treatment, the status of the CDK1-cyclinB1 complex was investigated. Asynchronously growing cells were incubated for 24 hours with the peptide fraction and the expression of cyclin B1 determined by flow cytometric analysis in G_2 _cells selected on the base of their DNA content. Fig. 6A shows that the level of cyclin B1 per cell is 1.5 higher in G_2_/M treated cells compared to the controls. We then analyzed whether the CDK1-cyclin B1 was in the active status by measuring its kinase activity. Synchronized cells were incubated with the chromatin peptide fraction at 1 hour after removal of thymidine block. They were harvested in the G_2 _phase (at 8 h) and the CDK1-cyclin B1 complex was purified from nuclear extracts. Despite the higher level of CDK1-cyclin B1 expression, its kinase activity was 3.4 times lower in treated cells compared with controls (Fig. 6B). This suggests that the peptide chromatin fraction causes inactivation of CDK1-cyclin B1 complex in G_2 _cells. To support these data the expression level of the inactive phospho-Tyr15 cdc2 in the same extract was evaluated. In Fig. 6C it is shown that it is effectively higher (1.5 times) in the treated cells compared to the controls (line a).

### Involvement of the G_2 _checkpoint control in the cell cycle arrest induced by chromatin peptides

Many authors have reported that DNA damage induces G_2 _arrest by activation of a molecular circuit, known as the G_2 _checkpoint control pathway which monitors DNA integrity prior to mitosis at the G_2_/M transition. DNA damage activates the DNA-PK/ATM/ATR kinases which phosphorylate chk1 at Ser 345, chk2 at Thr 68 and p53. The activated chk kinases inactivate cdc 25 phosphatase via phosphorylation at Ser 216. This blocks the activation of CDK1-cyclin B1 kinase and the progression into mitosis [22]. Since cells exposed to the peptide fraction showed both DNA damage and G_2 _arrest, the induction of this pathway was hypothesized to explain the mechanism of action of the peptides. The activation of the G_2 _checkpoint control was investigated by examining the activation of the chk1 kinase in G_2 _cells. Fig. 6C (line b) shows the expression of the active Chk1 kinase measured in nuclear extract carried out, as described above, in synchronized cells 8 hours after removal of the thymidine block. It results in 1.7 times more chk1 expression in treated cells compared to the controls. This is consistent with the increase of expression of the inactive CDK1-cyclin B1 complex and the decrease of its kinase activity observed after the treatment. These results support the hypothesis that DNA damage, induced by exposing HeLa cells to the pool of chromatin peptides, results in G_2 _arrest via the G_2 _checkpoint control pathway.

## Discussion

A class of small peptides that remains associated with extensively deproteinized DNA of eukaryotic and prokaryotic cells has previously been described in the literature. The biological activity of these peptides shows that they might represent a novel factor, shared by prokaryotic and eukaryotic cells, and active in the regulation of gene expression and cell growth. Interestingly, tumor cells express a lower level of this class of peptides as compared to the corresponding normal cells, therefore we hypothesized a role for them in the prevention of cellular transformation. Similar characteristic features have been reported for another phylogenetically ancient system of peptidergic regulation based on a pool of peptides derived from the proteolysis of functional proteins [23,24]. They too, might be involved in the maintenance of tissue homeostasis and hence, in carcinogenesis and tumor cell growth. This pool is tissue specific but species un-specific. Lastly, a series of small endogenous peptides that are N-substituted at the N-terminal end has been described as factors with a modulating effect on cell proliferation and differentiation: the hemoregulatory pentapeptide [25]; the epidermal pentapeptide [26]; the colon mitosis inhibitor [27] and the hepatic pentapeptide [28]. They show tissue specificity and can inhibit even the growth of malignant cells derived from the tissue organ from which they were isolated. Common to all three groups of peptides is that at present their target and the molecular mechanism of action are almost unknown.

In the present study, we wanted to provide some insight into the role of the afore-mentioned pool of chromatin peptides in inhibiting the proliferation of tumor cells. The results revealed that peptides isolated from wheat bud chromatin inhibit HeLa cells growth in a dose and time dependent manner. This effect is not due to general toxicity, but to the induction of the apoptotic process. The association of this class of peptides with the DNA and its ability to affect DNA and RNA synthesis in cell free systems suggests that it brings about a defective DNA template activity. Maximum inhibition of cell growth is obtained when the cells are treated during the S phase and it is accompanied by DNA single strand breaks and DNA synthesis inhibition. Eukaryotic cells have developed checkpoint control pathways for sensing DNA lesions to ensure a correct transmission of genetic information [29]. The activation of these pathways induces an arrest of the cell-cycle progression that allows time for removing defects or eliminating damaged cells. While normal cells, when exposed to genotoxic agents, are capable of arresting in G_1 _and G_2 _phases, most cancer cells have lost this ability. The possibility of recovering these functions might be a useful strategy in controlling their growth. We have shown that treatment for 12, 24 and 48 hours with 5 μg/ml peptide concentration results in an increase of G_2_/M cell number accompanied by a decrease in the percentage of cells in S phase. The distribution of the cells in the different phases of mitosis showed no accumulation of cells in any mitotic phase. This indicates that the cell cycle block occurs in G_2 _before the entry of the cell into mitosis. These results are consistent with the reported data on DNA damage and DNA synthesis inhibition. Thus, many authors have reported that genotoxic agents induce an arrest of the cell cycle in G_2 _phase [30,31]. A key element in controlling the progression to mitosis is the CDK1-cyclin B1 complex, a kinase conserved in all eukaryotes, which is therefore the target of most antiproliferative signals generated during S and G_2 _phase. Its activity is maintained at low levels during G_1 _and S phases, while a rapid increase is required for entry into mitosis. Several mechanisms are responsible for the regulation of CDK1-cyclin B1 activity during cell cycle: the synthesis of cyclin B1 to form the active complex, the inhibitory phosphorylation in the active site of the enzyme and the expression of inhibitors. Our data show that cyclin B1 accumulates in G_2 _treated cells while the CDK1-cyclin B1 kinase activity level is lower in comparison to the controls. This suggests that treated cells fail to activate this complex.

DNA damage or incomplete DNA replication results in the inhibition of this kinase activity via the activation of the G_2 _checkpoint control. This pathway prevents mitotic entry in the presence of genomic defects and ensures the proper transmission of genetic information from a cell to its daughters. In eukaryotic cells the ataxia-telangiectasia mutated (ATM) and ATM-related (ATR) protein kinases are activated in response to DNA damaging agents such as ionizing radiation, ultraviolet light and DNA replication inhibitors. They activate chk1 and chk2 protein kinases responsible for the inhibition via phosphorylation of the cdc25 phosphatase. It mediates the removal of the inhibiting phosphorylations on Tyr15 and Thr14 within the CDK1 ATP-binding domain. Consequently, activation of chk1 and chk2 leads to the inhibition of CDK1-cyclinB1 complex, which results in cell-cycle arrest prior to mitosis. We have shown that the treatment of the cells in S phase induces in G_2 _phase cells an increase of the expression of the active Chk1 kinase and of the inactive phospho-Tyr15 CDK1 kinase in G_2 _phase cells. This strongly indicates that the G_2 _checkpoint control pathway is involved. p53's role in the inhibition of the CDK1-cyclin B1 kinase complex at the G_2 _checkpoint has been reported [32]. However cells lacking p53 are shown to be capable of a G_2 _arrest by activating the part of the pathway that is p53-independent [33]. HeLa cells are p53 negative because they express HPVE6 protein which leads to the rapid degradation of p53 [34]; therefore it is likely that the observed G_2 _arrest is due to the activation of the p53-independent checkpoint control pathway. Cells which have initiated a G_2_/M checkpoint pathway in response to DNA damage can undergo a variety of fates including a prolonged arrest, re-entry into the cell cycle after DNA repair, apoptosis or progression through the cell cycle without repairing the DNA damage responsible for the arrest. In our system G_2 _arrest is accompanied by apoptosis, so it is quite likely that there is a close relationship between the two processes. This is consistent with a quantity of evidence by other authors showing that inactivation of CDK1 is related to apoptosis induction [35]. When we counted the mitotic phases we also found many cells with apoptotic morphology in G_2 _phase. Flow cytometry analysis revealed that most of the cells stained by TdT had a double DNA content. These findings suggest that, while a certain fraction of the cells in G_2 _are quite unaffected and continue through G_2 _and M, a number of cells that are blocked in G_2 _undergo apoptosis. The apoptosis pathway has been shown to be caspase dependent and to involve the activation of Bax, a likely consequence of the inability of the arrested cells to repair the genomic defects and to re-enter the cell cycle. On the basis of the data shown here, we assume that the pool of peptides inhibits HeLa cell proliferation by causing DNA damage or incomplete DNA replication which, in turn, activates the G_2 _checkpoint pathway that arrests the cell prior to mitosis. Preliminary results show that the cells that survive peptide treatment undergo G2 arrest also after the removal of the peptides from culture medium, thus suggesting that the effects on cell cycle progression are not reversible. These effect seem to be specific for these sequences since some DNA binding peptides (pyroGlu-Ala-Glu-Ser-Asn, pyroGlu Ala-Gly-Glu-Ser-Glu-Asp, pyroGlu-Ala-Gly-Glu-Glu-Ser-Asn, pyroGlu-Ala-Gly-Glu-Glu-Glu-Glu-Ser-Asn) and some known biologically active peptides (thymosin α_1 _fragment 25–28, serum thymic factor), used as control, didn't reproduce the regulatory activity on cell growth shown by the native chromatin peptides [36].

In a previous study the structural characterization of two peptide sequences belonging to the pool of this class of peptides extracted from trout testis chromatin active in inhibiting DNA transcription was reported. These structures [pyroGlu-Ala-Gly-Glu-Asp-Ser(P)-Asp-Glu-Glu-Asn and pyroGlu-Ala-val-Glu-Asp-Ser(P)-Asp-Glu-Asp-Thr] or very similar sequences are present in the portion corresponding to the phosphorylation site for CKII kinase of many proteins transcription factors such as RNA pol.II Sub.215 KD, PML, HPV18E7, Human Myc, Chicken Myb, Human Rfx, human Ubf1, RNA Pol. Sigma 70 factor [36]. Interestingly, phosphorylation of some of these nuclear proteins (RNA polymerase, Myb) by CkII regulates the proteins' function and DNA binding ability. Furthermore, the phosphorylation sequence in PML and Myb proteins is deleted following oncogenic activation. These observations indicate that the molecular models proposed for some of the sequences forming our pool of peptides could: i) play a role in DNA replication/transcription activity ii) open for the possibility that the pool of peptides could be generated from a proteolytical process of non-histone proteins, since chromatin bound proteases have been reported by several authors [37]. However, synthetic peptides, synthesized on the basis of the proposed models, exhibit effects on RNA synthesis in cell free systems quite similar to those obtained with the native fraction, but were inactive at cellular level. These data suggest that although the proposed sequences might be effectors present in the family of the native peptides, further structural analyses are needed to explain the reported effects on cell growth. We also speculate that the inability of the proposed structures to affect cell proliferation is due to their poor bio-availability for cell treatment and that they are present in the native pool in a complexed form which is more effective for cell interaction.

Studies are in progress to better clarify the structure-function relationship of chromatin peptides by including further steps in their purification procedure and by improving HPLC and mass spectrometry analysis experiments. The overall goal is to obtain different molecular models of peptide sequences.

## Conclusion

In conclusion we have found a new class of DNA-binding peptides that are able to inhibit the cell cycle by inducing DNA damage, and to promote apoptosis in HeLa cells. The reported regulatory effects on the growth of a tumor cell line are consistent with their ubiquitous presence in normal cells and their lower level in cancer cells and thus support the hypothesis that they are relevant in carcinogenesis. Further studies to understand their involvement in cell proliferation may be helpful in elucidating neoplastic transformation.

## Methods

### Purification of peptides from wheat bud chromatin

The isolation of peptides from plant tissues has been already described in detail [6]. Briefly, the chromatin was purified at slightly acidic pH and then subjected to alkaline extraction by adding ammonia to pH 9.5. The macromolecules were precipitated with two volumes of methanol and removed by centrifugation. The peptide component was purified from the supernatant by ion exchange and gel filtration chromatography.

### Cell culture

HeLa cells (from ATCC cell lines, catalogue number CCL-2) were tested for mycoplasms by using Mycoplasma Detection Kit (Roche Germany). They were grown in 25 cm^2 ^flasks with 5 ml of Dulbecco's Modified Eagle Medium with 10% fetal calf serum, penicillin at 100 units/ml, streptomycin at 100 μg/ml and 2 mM glutamine (Invitrogen Paisley UK) at 37°C humidified atmosphere of 5% CO_2_/95% air. The peptides were added to the culture medium 24 hours after cell seeding (25000 cells/cm^2^). Cell numbers were monitored by direct counting using a hemocytometer. Cell viability was assessed by trypan blue exclusion and by the determination of lactic dehydrogenase activity in the culture medium. The enzyme assay was performed by incubating different amount of culture medium with 0.2 M K-phosphate buffer pH 7.6 containing 0.2 mM pyruvic acid and 0.2 mM NADH. All chemicals were obtained from Sigma-Aldrich corp. (St Louis MO). The reaction was followed by measuring the absorbance decrease of NADH at λ = 340 nm. All the experiments with cells were performed in triplicate.

### Cell synchronization by double thymidine block

HeLa cells in logarithmic growth (10000/cm^2 ^cell density) were synchronized by a double thymidine block as described by Kozaki et al [18]. After seeding, they were allowed to grow for 6 h, then the medium was removed and the cells were exposed successively to 2 mM thymidine (Sigma) for 16 h, to normal medium for 8 h, and to 2 mM thymidine for 16 h. The subsequent replacement with normal medium released the cells at the G_1_/S boundary.

### Incorporation of ^3^H-thymidine into DNA

Synchronized cells were grown at 10000/cm^2 ^cell density and ^3^H-thymidine incorporation was determined at different time points after the removal of the thymidine block by incubating the cells with 1 μCi/ml of ^3^H-thymidine (Amersham, Buckinghamshire, UK) for 1 h. Then, the radioactive medium was removed, the cells fixed in methanol for 10 min, washed twice with 10% trichloroacetic acid for 10 min and solubilized with a solution of 1% trichloroacetic acid and 0.3 N NaOH. Incorporation of radioactive thymidine was measured by liquid scintillation counting.

### Cell cycle analysis

Trypsinized cell suspensions were fixed in 80% ethanol. The samples were then placed at -20°C until cell cycle analysis. Nuclei were isolated from fixed cell suspensions using Vindeløv's protocol [38] stained with propidium iodide (50 μg/ml), and samples analyzed for DNA content (PI red fluorescence) using a FACSCalibur laser flow cytometer (Becton Dickinson Immunocytometry Systems, San Jose, CA). Pulse-processed fluorescence signals were used to exclude doublets and aggregates from analyses. Ten thousand events were acquired for each sample. Percentages of cells in the G_1_, S, and G_2_M phases of the cell cycle were quantified using WinCycle software (Phoenix Flow Systems, San Diego, CA).

### Quantification of apoptosis

Apoptotic response to peptide exposure was quantified using the TUNEL method for apoptosis detection. Briefly, apoptotic DNA fragments in control and treated samples were end-labeled with biotin-conjugated dUTP via terminal deoxynucleotidyl transferase (TdT) and detected using streptavidin-FITC [39]. Cells were counterstained with 5 μg/ml propidium iodide to stain cellular DNA. The resulting labelled apoptotic fractions were quantified in control and treated cell samples using a FACSCalibur laser flow cytometer (BDIS, San Jose, CA), after appropriate gating using pulse-width analysis of the DNA content signal to exclude doublets and aggregates.

### ELISA detection of cytoplasmic nucleosomes

Apoptosis levels were measured in exponentially growing cells using the cell death detection ELISA kit (Boehringer Mannheim Ge), following the instructions of the manufacturer. The photometric enzyme-immunoassay allows in vitro quantification of mono- and oligonucleosomes in the cytoplasm derived from apoptosis. The results are expressed as percentage of optical density, resulting from the activity of the peroxidase-conjugated anti-DNA antibody complexed with cytoplasmic nucleosomes of treated cells, compared with the control.

### Detection of cell cycle regulatory proteins using flow cytometry

Cells (2 × 10^6^) were trypsinized, washed twice with 10 mM phosphate buffer (PBS) and fixed in 90% ethanol. After washing in PBS the pellet was treated with PBS plus 0.1% Triton X and resuspended in 100 μl PBS containing 0.1% Triton X100 plus 3% non-fat dry milk. Primary antibodies (cdc2, cyclin B1, Santa Cruz Biotechnology) were added to a final concentration of 2 μg/ml, and allowed to incubate on ice for 1 hour. Suspensions were then washed in PBS containing 0.1% Triton X100. Pellets were resuspended and incubated with PBS containing 0.1% Triton X100, 3% non-fat dry milk, and biotinylated horse-anti-mouse IgG (1:50 dilution of stock, Vector Labs, Burlingame, CA) for 30 minutes. Suspensions were then washed in PBS containing 0.1% Triton X100. Pellets were resuspended and incubated with PBS containing 0.1% Triton X100, 3% non-fat dry milk, and streptavidin-FITC (1:50 dilution of stock, Amersham Biosciences, UK) for 30 minutes. Suspensions were again washed in PBS containing 0.1% Triton X100, and then resuspended in 5 μg/ml propidium iodide made up in PBS containing 0.1% Triton X100, to stain DNA. Samples were then run on a FACSCalibur flow cytometer, and correlated analyses of cell cycle proteins and DNA content (to allow for cell cycle analysis) were generated.

### Preparation of proteins from nuclear extracts

Nuclei purification was performed from exponentially growing HeLa cells as previously described [40]. The cells, harvested by trypsinization and washed twice with PBS, were kept for 8 min at 4°C in a solution (1 ml/2 × 10^7 ^cells) containing 10 mM Tris pH 7.4, 10 mM NaCl, 3 mM MgCl_2_, 3 mM DTT and 0.5% Nonidet P-40. An equal volume of 1.4 M sucrose was added and the resulting suspension centrifuged at 11000 rpm for 10 minutes (microcentrifugette ALC 4214 rotor A-12). The nuclei contained in the pellet were resuspended in a sample buffer solution (1 ml/2 × 10^7 ^cells) containing 50 mM TRIS HCl pH 7.5, 0.5 M NaCl, 5 mM EDTA, 2 mM EGTA, 0.01% Brij35, 50 mM β-mercaptoethanol, 1 mM PMSF, 0.05 mg/ml leupeptin, 1 mM Na-orthovanadate and 25 mM β-glycerophosphate. Nuclear proteins were recovered in the super after centrifugation at 11000 rpm for 1 hour (microcentrifugette ALC 4214 rotor A-12) and used for SDS-PAGE.

### SDS-PAGE and immunoblotting

The proteins (40 μg) were separated by SDS-PAGE on 12.5% acrylamide gels according to Laemmli [41] and were transferred to nitrocellulose filters by using 25 mM Tris, 192 mM glycine, 20% methanol at 100 V for 1 h, according to Towbin et al [42]. Nitrocellulose sheets were incubated overnight with anti-phospho-CDK1 (Tyr15), anti-phospho-chk1 (Ser345), anti-phospho-Bcl-2 rabbit polyclonal IgG antibodies and with anti-Bax, anti-cleaved caspase-7, anti-cleaved caspase-9 and anti-cleaved PARP rabbit polyclonal IgG antibodies. They were then incubated with a secondary anti-rabbit IgG HRP-linked antibody. All antibodies were obtained from Cell Signaling (Beverly, MA) and diluted following the instructions of the manufacturer. The blots were then developed with LumiGLO chemiluminescent reagent (Sigma) and the blot bands quantified using the NIH Image densitometry program.

### CDK1 kinase assay

CDK1 activity was measured by MESACUP cdc2 Kinase Assay Kit (MBL, Japan) following the manufacturer instructions. The kit is based on ELISA assay that utilizes a synthetic peptide as substrate and a monoclonal antibody recognizing the phosphorylated form of the peptide. Incorporation of radioactive thymidine was measured by liquid scintillation counting.

### Single cell gel electrophoresis assay (Comet assay)

Cultured cells were harvested by trypsinization and washed twice with PBS. They were then dispersed and immobilized in LMP agarose gel on microscope slides. The detection of comet formation was carried out under different pH conditions. In the alkaline comet assay [21] the slides were placed in a lytic solution (2.5 M NaCl, 100 mM Na_2_EDTA, 10 mM TRIS, 10% DMSO 1% Triton X 100 pH 10) for 1 h at 4°C. After 20 min of incubation in electrophoresis buffer (0.3 M NaOH, Na_2_EDTA 1 mM) the slides were subjected to electrophoresis for 20 min at 25 V and 300 mA. Following electrophoresis the slides were rinsed in neutral buffer (0.4 M TRIS-HCl pH 7,5) and stained with ethidium bromide to examine the DNA migration. Neutral comet assay was performed as described in [43]. The slides were placed in a lysing solution consisting of 30 mM EDTA, 0.5% SDS, pH 8.0 at 50°C for 4 h. They were then washed in TBE buffer (90 mM TRIS, 2 mM EDTA, 90 mM boric acid, pH 8.5) for 16 h and then subjected to electrophoresis in TBE buffer at 25 volts for 20 min. The slides were rinsed and stained as above. Tail moment was calculated using Comet assay II image analysis system (Perceptive Instrument UK) fitted with a fluorescence microscope.

## Competing interests

The authors declare that they have no competing interests.

## Authors' contributions

LM designed the experiments and drafted the manuscript. PMD performed the flow cytometric analyses and was involved in revision and final approval of the manuscript. LA and VP carried out the experiments with cells and participated in the design of the study. KE was involved in manuscript revision and provided critical insight. GG supervised the project and commented on the manuscript.
